# Case Report: Influenza B-triggered CIDP revealing PMP22-related Dejerine–Sottas-like neuropathy in a child—biphasic cytokine dynamics and response to immunotherapy

**DOI:** 10.3389/fmed.2026.1841187

**Published:** 2026-07-15

**Authors:** Yazhen Fan, Junying Qiao, Jingpo Zhang, Jianchuang Zhao, Xianjie Huang, Chenhang Cui, Na Wang

**Affiliations:** Department of Pediatric Intensive Care Unit, The Third Affiliated Hospital of Zhengzhou University, Zhengzhou, China

**Keywords:** chronic inflammatory demyelinating polyradiculoneuropathy, cytokine dynamics, Dejerine-Sottas disease, immunotherapy, influenza B infection, PMP22

## Abstract

Dejerine–Sottas disease (DSD) is a severe early-onset hereditary demyelinating neuropathy, but its overlap with chronic inflammatory demyelinating polyradiculoneuropathy (CIDP) has rarely been described in children. We report a child in whom influenza B-triggered CIDP led to the recognition of a previously undiagnosed PMP22-related hereditary demyelinating neuropathy with a severe Dejerine–Sottas-like phenotype. The patient had longstanding developmental delay and neuropathic features before the acute illness. After severe influenza B infection, he developed progressive weakness lasting more than 8 weeks, marked albuminocytologic dissociation, biphasic cytokine elevation in serum and cerebrospinal fluid (CSF), diffuse peripheral nerve enlargement, and electrophysiological evidence of diffuse, largely symmetric sensorimotor polyneuropathy with prominent demyelinating features and secondary axonal involvement. Genetic testing identified a maternally inherited heterozygous 1.38-Mb deletion at chromosome 17p12 involving the dosage-sensitive PMP22 gene. Treatment with intravenous immunoglobulin and corticosteroids was followed by improvements consciousness, respiratory function, limb strength, and cytokine levels, although motor recovery remained incomplete. In children with features of hereditary neuropathy, acute or subacute deterioration after infection should prompt evaluation for superimposed CIDP, as the inflammatory component may be responsive to immunotherapy.

## 1 Introduction

DSD is a severe early-onset hereditary demyelinating neuropathy within the Charcot–Marie–Tooth (CMT) spectrum, usually presenting in infancy with delayed motor milestones, hypotonia, areflexia, distal weakness, muscle wasting, sensory impairment, and foot deformities (1). Peripheral myelin protein 22 (PMP22) is among the genes most commonly implicated in DSD (1). CIDP is an immune-mediated demyelinating neuropathy characterized by a disease course exceeding 8 weeks, sometimes triggered by infection-induced immune-inflammatory activation, characterized by presentation as either a monophasic or a relapsing–remitting disorder, and potential responsiveness to immunotherapy (2). Although pediatric CIDP is increasingly recognized, its overlap with hereditary demyelinating neuropathy remains uncommon and diagnostically challenging (3, 4). We report a child in whom severe influenza B infection was followed by CIDP, leading to the recognition of a previously undiagnosed PMP22-related hereditary demyelinating neuropathy with a severe Dejerine–Sottas-like phenotype. The case emphasizes the need to consider superimposed inflammatory neuropathy when post-infectious deterioration occurs in children with longstanding neuropathic features. Written informed consent for publication was obtained from the patient’s legal guardians.

## 2 Case description

### 2.1 Preexisting developmental and neuropathic features

A 9-year-old boy had longstanding developmental and neuropathic abnormalities before the current illness. He had delayed motor milestones, steppage gait, frequent falls, inability to run, poor hand function, and dependence on caregivers for feeding. He had no spoken words or meaningful verbal expression. Despite previous rehabilitation, functional improvement was limited. At baseline, the Hughes functional grade was 2–3, and the Overall Neuropathy Limitations Scale (ONLS) score was 6, with no family history of similar disorders.

### 2.2 Influenza B infection and systemic inflammatory phase

In November 2023, the patient developed vomiting and abdominal distension following diarrhea, which improved, but progressive limb weakness developed. After 45 days of persistent weakness despite treatment, he was transferred to our pediatric intensive care unit. One day before admission, he had a fever of 40 °C and was comatose on arrival. Examination showed cachexia, muscle wasting, ulnar claw hands, pes cavus, hammer toes, absent tendon reflexes, and muscle strength of grade II with generalized hypotonia. Respiratory distress led to endotracheal intubation and mechanical ventilation. Laboratory tests revealed elevated levels of CK-MB (253 U/L), CK (886 U/L), creatinine (122 μmol/L), amylase (278 U/L), lipase (642 U/L), and ALT (272 U/L). Serum cytokines were markedly elevated, including IL-10 (397.5 ng/L), IL-6 (565.9 ng/L), and IL-8 (1360.8 ng/L). CSF analysis showed 2 × 10^6^/L leukocytes and protein of 575 mg/L, with negative pathogen testing. Serum influenza B nucleic acid and IgM were positive. Chest radiography showed scoliosis, and electroencephalography showed low-amplitude slow waves. The clinical picture was consistent with influenza B infection, sepsis, and influenza B-associated encephalopathy in the context of systemic inflammation. Treatment with methylprednisolone and IVIG led to recovery of consciousness and normalization of laboratory abnormalities by day 10; muscle strength improved to grade 4, and ventilatory support was discontinued on day 15 with transition to nasal cannula oxygen. After methylprednisolone, IVIG, anti-infective treatment, and supportive care, consciousness recovered and laboratory abnormalities improved by illness day 55. Muscle strength improved to grade 4. Mechanical ventilation was discontinued on illness day 60 with transition to nasal cannula oxygen.

### 2.3 Post-infectious CIDP phase and response to immunotherapy

After the initial improvement, the patient developed a second phase of neurological deterioration. On illness day 67, while fully conscious, the patient developed progressive symmetric weakness from proximal to distal muscles, accompanied by hyperhidrosis, impaired pain sensation, and shallow breathing. Re-intubation and mechanical ventilation were required. On illness day 73, repeat CSF analysis showed 2 × 10^6^/L leukocytes and markedly increased protein of 5,500 mg/L, consistent with albuminocytologic dissociation. Muscle strength declined to grade 0–1 in all limbs, with areflexia and diffuse sensory loss, with no cortical or cranial nerve involvement. Brain magnetic resonance imaging (MRI) showed no central nervous system (CNS) demyelinating lesions. Serum and CSF testing was negative for antiganglioside, nodal/paranodal, and CNS demyelinating antibodies. Functional disability worsened to Hughes grade 5 and ONLS 12. Serum cytokines increased again, including IL-2 (97.4 ng/L), IL-6 (214.6 ng/L), IL-8 (368.1 ng/L), IL-10 (16.9 ng/L), TNF-*α* (17.9 ng/L), TNF-*β* (34.73 ng/L), IFN-*γ* (99.4 ng/L), and IL-12p70 (37.2 ng/L). CSF IL-8 increased to 237.3 ng/L. CSF albumin was 3,850 mg/L, CSF IgG was 820.0 mg/L, the IgG index was 1.4, and 24-h intrathecal IgG synthesis was 168 mg/24 h; oligoclonal bands and serum immunoglobulins were normal. Peripheral nerve ultrasonography showed diffuse, symmetric enlargement and reduced echogenicity of the bilateral ulnar, median, common peroneal, and tibial nerves, with maximum cross-sectional areas of 15.2 mm^2^, 13.5 mm^2^, 12.8 mm^2^, and 9.2 mm^2^, respectively.

**Figure 1:**
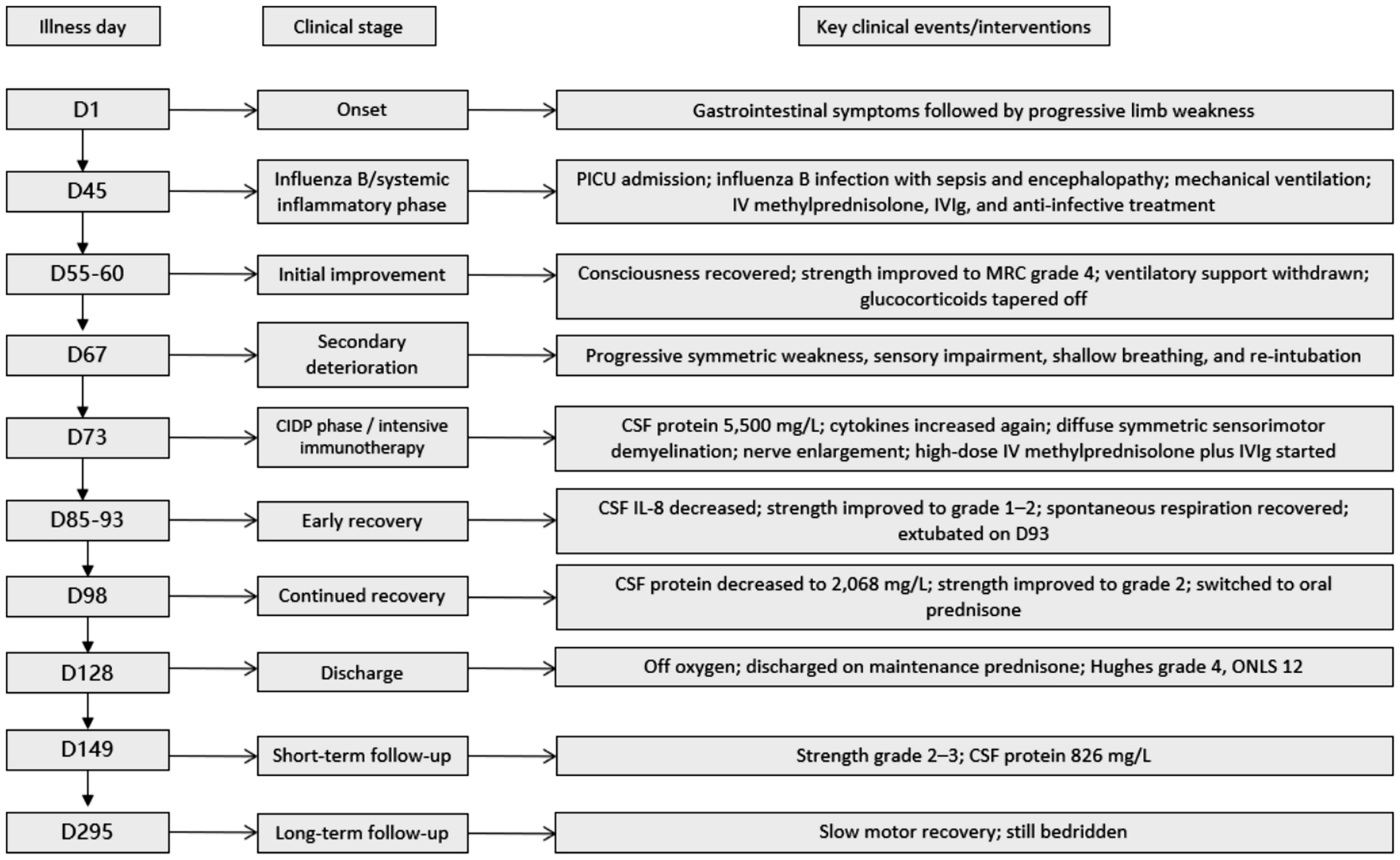
Diagnostic and treatment flowchart for the pediatric patient.

**Figure 2:**
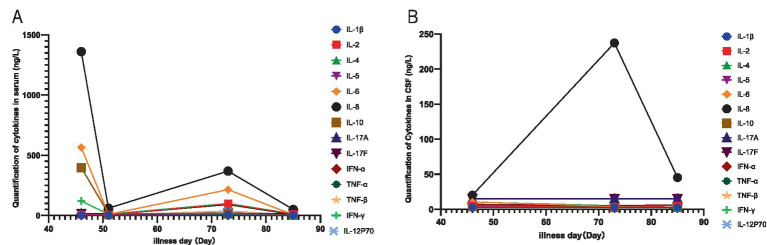
Temporal dynamics of serum and CSF cytokine levels across illness days. **(A)** Serum cytokine concentrations. **(B)** CSF cytokine concentrations. The cytokines upper reference limits were: IL-1β ≤3.4 ng/L, IL-2 ≤5.71 ng/L, IL-4 ≤2.8 ng/L, IL-5 ≤4.15 ng/L, IL-6 ≤5.3 ng/L, IL-8 ≤15.71 ng/L, IL-10 ≤4.91 ng/L, IL-12p70 ≤3.2 ng/L, IL-17A≤20.6 ng/L, IL-17F ≤20.3 ng/L, IFN-α ≤7.9 ng/L, TNF-α ≤4.6 ng/L, TNF-β ≤5.8 ng/L, and IFN-γ ≤15.49 ng/L.

An infection-triggered CIDP episode was suspected. High-dose intravenous methylprednisolone (20 mg/kg/day for 7 days) plus IVIg (0.4 g/kg/day for 5 days) was started on illness day 73. Ten days later, limb strength improved to grade 1–2, spontaneous respiration gradually recovered, and intermittent breathing training was started through the endotracheal tube. On illness day 93, the endotracheal tube was removed, and oxygen support was changed to a nasal cannula. Serum cytokines and CSF IL-8 declined after immunotherapy, with CSF IL-8 decreasing to 45.2 ng/L by illness day 85. Methylprednisolone was tapered and changed to oral prednisone at 1 mg/kg/day. On illness day 98, CSF protein decreased to 2,068 mg/L, and limb strength improved to grade 2. Nasal cannula oxygen was discontinued on illness day 128. The patient was discharged on maintenance prednisone with Hughes grade 4 and ONLS 12. Three weeks after discharge, limb strength improved to grade 2–3, and CSF protein decreased to 826 mg/L. At 6 months, motor recovery remained slow, and the patient was still bedridden. The clinical course, laboratory findings, treatments, and follow-up are summarized in Figure 1. Temporal changes in serum and CSF cytokines are shown in Figure 2.

### 2.4 Electrophysiological, family, and genetic evaluation

Detailed electrophysiological findings are summarized in Tables 1–3. On illness day 73, motor responses were absent in the tested left median, ulnar, deep peroneal, and tibial nerves, and sensory responses were absent in the tested left median, ulnar, sural, and superficial peroneal nerves. On follow-up studies, responses partially reappeared, but compound muscle action potential (CMAP) and sensory nerve action potential (SNAP) amplitudes remained reduced, conduction velocities were slowed, and F-wave responses remained absent. Needle electromyography showed widespread fibrillation potentials and positive sharp waves with reduced recruitment of motor unit action potentials (MUAPs). Follow-up testing showed persistent spontaneous activity with reduced MUAP recruitment (Table 3).

**Table 1:** Serial left-sided NCS parameters in the patient during the CIDP phase.

Illness day (d)	Motor nerve	Distal latency (ms)	CMAP amplitude (mV)	MNCV (m/s)	F-wave latency (ms)	Sensory nerve	Peak latency (ms)	SNAP amplitude (μV)	SNCV (m/s)
73	Median	NR	NR	NR	NR	Median	NR	NR	NR
	Ulnar	NR	NR	NR	NR	Ulnar	NR	NR	NR
	Deep peroneal	NR	NR	NR	NR	Sural	NR	NR	NR
	Tibial	NR	NR	NR	NR	Superficial peroneal	NR	NR	NR
108	Median	8.0 (prolonged)	0.6 (reduced)	44 (slowed)	NR	Median	2.8	5 (reduced)	36 (slowed)
	Ulnar	7.7 (prolonged)	0.5 (reduced)	44 (slowed)	NR	Ulnar	2.4	7 (reduced)	31 (slowed)
	Deep peroneal	NR	NR	NR	NR	Sural	2.4	7 (reduced)	42 (slowed)
	Tibial	NR	NR	NR	NR	Superficial peroneal	2.7	16 (reduced)	32 (slowed)
166	Median	6.2 (prolonged)	1.1 (reduced)	46 (slowed)	NR	Median	2.6	11 (reduced)	38 (slowed)
	Ulnar	5.1 (prolonged)	1.3 (reduced)	46 (slowed)	NR	Ulnar	2.5	9 (reduced)	34 (slowed)
	Deep peroneal	7.1 (prolonged)	0.8 (reduced)	39 (slowed)	NR	Sural	1.8	10 (reduced)	45 (slowed)
	Tibial	6.0 (prolonged)	0.6 (reduced)	35 (slowed)	NR	Superficial peroneal	1.6	18	36 (slowed)

CMAP, compound muscle action potential; SNAP, sensory nerve action potential; MNCV, motor nerve conduction velocity; SNCV, sensory nerve conduction velocity; NR, no response. Prolonged, reduced, and slowed indicate values outside laboratory reference ranges. Motor and sensory nerves are listed side by side for compact presentation and do not indicate paired recordings from the same nerve.

**Table 2:** Left-sided NCS parameters in the patient’s mother.

Motor nerves	Distal latency (ms)	CMAP amplitude (mV)	MNCV (m/s)	F-wave latency (ms)	Sensory nerves	Peak latency (ms)	SNAP amplitude (μV)	SNCV (m/s)
Median	3.6	10.5	57	26.5	Median	2.7	29	45 (reduced)
Ulnar	3.2	6.2	50 (reduced)	24.1	Ulnar	2.8	23	37 (reduced)
Deep peroneal	6.2 (prolonged)	4.2	42 (reduced)	54.2	Sural	2.6	10 (reduced)	39 (reduced)
Tibial	6.2 (prolonged)	6.9	41 (reduced)	52.2	Superficial peroneal	2.1	22	40 (reduced)

**Table 3:** Serial needle electromyography findings in the patient at different illness days.

Illness day (d)	Muscle	Insertional activity	Spontaneous activity – Fibrillation potentials	Spontaneous activity – Positive sharp waves	Spontaneous activity – Fasciculation potentials	MUAP recruitment
73	Tibialis anterior, R	Prolonged	1+	2+	0	Reduced
	Rectus femoris, R	Prolonged	1+	2+	0	Reduced
	Tibialis anterior, L	Prolonged	1+	2+	0	Reduced
	Rectus femoris, L	Prolonged	1+	2+	0	Reduced
	First dorsal interosseous, R	Prolonged	1+	4+	0	Reduced
	Biceps brachii, R	Prolonged	1+	4+	0	Reduced
	Flexor carpi radialis, R	Prolonged	1+	4+	0	Reduced
98	Tibialis anterior, R	Prolonged	2+	3+	0	Reduced
	Medial gastrocnemius, R	Prolonged	2+	3+	0	Reduced
	Tibialis anterior, L	Prolonged	2+	3+	0	Reduced
	Medial gastrocnemius, L	Prolonged	2+	3+	0	Reduced
	Rectus femoris, L	Prolonged	2+	3+	0	Reduced
	Flexor carpi radialis, R	Prolonged	2+	3+	0	Reduced
	Biceps brachii, R	Prolonged	2+	3+	0	Reduced

MUAP, motor unit action potentials; R, right; L, left. The muscles examined differed slightly between illness days.

Nerve conduction studies (NCS) in the patient’s asymptomatic 33-year-old mother showed sensory-predominant symmetric demyelinating abnormalities (Table 2), prompting trio-based genetic testing. Genetic analysis identified a maternally inherited heterozygous deletion of approximately 1.38 Mb at chromosome 17p12, chr17p12:14095286–15472395. The deleted interval involved the dosage-sensitive PMP22 gene and the spermatogenesis-related gene TEKT3, representing a contiguous gene deletion rather than an isolated sequence-level PMP22 variant. The father did not carry this deletion. According to ACMG criteria for copy-number variant interpretation, the deletion was classified as pathogenic.

Because PMP22 dosage alterations are established causes of hereditary demyelinating neuropathy, and because the mother carrying the same deletion had mild demyelinating abnormalities, the patient was diagnosed clinically as having DSD, corresponding to a PMP22-related hereditary demyelinating neuropathy with a severe Dejerine–Sottas-like phenotype. The inheritance pattern was consistent with autosomal dominant transmission with variable expressivity. The acute/subacute post-infectious deterioration, disease course exceeding 8 weeks, marked albuminocytologic dissociation, biphasic cytokine elevation, electrophysiological demyelination, peripheral nerve enlargement, and partial response to IVIg and corticosteroids were consistent with superimposed infection-triggered CIDP.

### 2.5 Caregiver perspective

As the patient had limited expressive language, his caregiver provided this perspective. The caregiver described the acute deterioration, respiratory failure, and prolonged hospitalization as a major burden for the family. After immunotherapy and supportive care, the family noticed improvements in consciousness, breathing, and limb movement. Recovery remained incomplete, and the child still required considerable assistance with daily activities.

## 3 Discussion

This case represents infection-triggered CIDP in a child with a previously unrecognized PMP22-related hereditary demyelinating neuropathy and a severe Dejerine–Sottas-like phenotype. Before the acute illness, the patient already had longstanding neuropathic features, including delayed motor development, distal weakness and wasting, scoliosis, pes cavus, hammer toes, areflexia, and steppage gait. These findings suggested an underlying hereditary neuropathy that had not been genetically defined. During the current episode, influenza B infection was followed by acute/subacute neurological deterioration, marked albuminocytologic dissociation, biphasic cytokine elevation, diffuse peripheral nerve enlargement, and partial response to IVIg and corticosteroids. Family screening and genetic testing later identified a maternally inherited heterozygous 1.38-Mb deletion at chromosome 17p12 involving the dosage-sensitive PMP22 gene. Together with the mother’s mild demyelinating abnormalities, these findings supported a PMP22-related hereditary demyelinating neuropathy with autosomal dominant inheritance and variable expressivity,with a severe Dejerine–Sottas-like phenotype (1). Thus, the inflammatory episode led to recognition of the underlying hereditary neuropathy, rather than occurring in a child with an established genetic diagnosis.

CIDP is an immune-mediated demyelinating peripheral neuropathy that is relatively uncommon in the pediatric population (3–5). It typically follows a chronic progressive, monophasic, or relapsing–remitting course lasting more than 2 months, may be preceded by upper respiratory or gastrointestinal infection, and typically shows demyelinating abnormalities on electrophysiological studies (5, 6). According to the 2021 European Academy of Neurology/Peripheral Nerve Society (EAN/PNS) criteria, CIDP is characterized by progression beyond 8 weeks. Electrophysiological criteria require demyelinating abnormalities in at least two motor nerves, together with reduced conduction velocity or decreased sensory nerve action potential amplitudes in at least two sensory nerves. When demyelination is demonstrated in only one nerve, at least two supportive criteria are required, including objective improvement after immunotherapy, CSF albuminocytologic dissociation, enlargement of two or more limb nerves or nerve plexus sites on ultrasound or MRI, and sural nerve biopsy findings consistent with CIDP. Other causes of similar peripheral neuropathies should also be excluded (7). In this patient, the disease course lasted more than 8 weeks and was accompanied by marked albuminocytologic dissociation, diffuse electrophysiological abnormalities, symmetric nerve enlargement on neurosonography, and partial response to IVIg and corticosteroids, supporting infection-triggered CIDP. Guillain–Barré syndrome was an important initial consideration, given the preceding gastrointestinal symptoms and influenza B infection, followed by weakness, areflexia, and respiratory failure. However, several features argued against a monophasic Guillain–Barré syndrome or treatment-related fluctuation (8, 9), including progression beyond the acute phase, recurrent deterioration after initial improvement, very high CSF protein level, persistent abnormalities on serial electrophysiological studies, and responsiveness to corticosteroids. Recurrent Guillain–Barré syndrome was also unlikely because the patient did not have discrete episodes separated by complete or near-complete recovery. Acute-onset CIDP was also relevant in the differential diagnosis, as its early course may closely resemble Guillain–Barré syndrome. In this patient, the disease course extending beyond 8 weeks, together with the need for continued immunotherapy, was more consistent with CIDP (10). ICU-acquired weakness and critical illness neuropathy were also less likely, as they could not explain the marked albuminocytologic dissociation, symmetric nerve enlargement, pre-existing skeletal deformities and distal muscle wasting, or the inherited PMP22-related neuropathy (6). Other inflammatory neuropathies were considered less likely because antiganglioside, nodal/paranodal, and CNS demyelinating antibodies were negative, and brain MRI showed no evidence of CNS demyelination.

The coexistence of a PMP22-related hereditary demyelinating neuropathy and superimposed CIDP likely contributed to the severe presentation and limited recovery. Although the role of immune mechanisms in Dejerine-Sottas-like phenotypes remains unclear, structural abnormalities in myelin proteins associated with DSD may render peripheral nerves more vulnerable to secondary immune-mediated injury after systemic infection, and post-infectious immune activation directed against peripheral myelin components may have been superimposed on the pre-existing genetic defect, thereby aggravating demyelination and secondary axonal injury (11). Chronic myelin instability, exposure of nerve antigens, and inflammatory cell infiltration around peripheral nerves may amplify local immune activation and aggravate demyelination (11). In patients with both hereditary neuropathy and acquired immune inflammation, while systemic infection serves as an external trigger that transforms subclinical inflammation into overt immune-mediated demyelination, these mechanisms may overlap, leading to combined demyelinating and secondary axonal injury, ultimately leading to CIDP superimposed on DSD (12). In this patient, Serial electrophysiological studies showed diffuse, largely symmetric sensorimotor involvement with prominent demyelinating features and secondary axonal injury. In the earliest study, motor and sensory responses were absent, which limited assessment of conduction block and temporal dispersion. Follow-up studies showed partial reappearance of responses with reduced amplitudes, slowed conduction velocities, and persistent absence of F waves, while needle EMG showed ongoing active denervation and reduced recruitment, consistent with severe peripheral nerve involvement and incomplete electrophysiological recovery. Neurosonography also showed diffuse, symmetric enlargement and hypoechogenicity of multiple limb nerves, consistent with widespread peripheral nerve involvement. Together with the electrophysiological findings, this pattern suggests that the pre-existing hereditary neuropathy may have increased nerve susceptibility, while influenza B infection and systemic inflammation precipitated the acquired immune-mediated deterioration (13).

Overlap between hereditary neuropathies and CIDP has been described, although pediatric cases remain rare. To our knowledge, infection-triggered CIDP revealing underlying DSD in a child has not been well documented. Escorcio-Bezerra et al. (11) described an adult patient with childhood-onset CMT who developed superimposed CIDP following varicella-zoster virus infection, and presented with progressive quadriplegia over 6–10 weeks. Campagnolo et al. (13) reported nine patients diagnosed with CIDP who carried pathogenic variants in PMP22, MPZ, or EGR2, including two with PMP22 duplication, these patients had elevated CSF protein levels (up to 1,500 mg/L) and electrophysiological features of sensorimotor demyelinating polyradiculoneuropathy with secondary axonal involvement; four showed partial responses to IVIg or corticosteroids, further supporting the coexistence of hereditary and inflammatory neuropathies and highlighting the phenotypic overlap between certain CMT subtypes and CIDP. Cardellini et al. (14) described a patient with subclinical MPZ-related CMT1B who developed overt symptoms after superimposed CIDP and achieved remission with long-term IVIg therapy. Conversely, Fernandez-Garcia et al. (15) identified PMP22- or MPZ-related neuropathies in patients initially diagnosed with CIDP who responded poorly to IVIg, emphasizing the value of genetic reassessment in atypical or treatment-refractory cases. The present case adds a pediatric example in which influenza B-triggered immune-inflammatory activation may have occurred in a pre-existing PMP22-related hereditary demyelinating neuropathy, resulting in superimposed CIDP with severe demyelination and secondary axonal injury. Although IVIg and corticosteroids were followed by partial clinical improvement, secondary axonal injury probably contributed to slow motor recovery and persistent disability.

The biphasic cytokine changes were in keeping with post-infectious immune activation during the CIDP phase, although they should not be taken as evidence of causality. During the septic phase, serum IL-6, IL-8, IL-10, and IFN-*γ* were markedly elevated, consistent with systemic inflammatory activation during severe infection, and declined after infection control. During the CIDP phase, serum IL-2, IL-6, IL-8, and IL-10 increased again, accompanied by a marked rise in CSF IL-8, indicating a biphasic cytokine pattern more pronounced in serum than in CSF. After IVIg and high-dose corticosteroids treatment, neurological status improved, serum cytokines largely normalized, and CSF IL-8 declined markedly. Previous studies have demonstrated immune-cell infiltration, including T and B lymphocytes, inflammatory cytokine activation, and macrophage-mediated myelin phagocytosis in CIDP, supporting the role of immune-mediated inflammation in demyelination and treatment responsiveness to immunotherapy (11–14). Autoantibodies against peripheral nerve antigens, including nodal or paranodal proteins such as NF155, have also been reported in a subset of patients within the CIDP spectrum (3, 16–18), In this patient, serum and CSF testing for antiganglioside, nodal/paranodal, and cerebrospinal fluid demyelinating antibodies was negative, and no antibody-mediated mechanism could be demonstrated. The biphasic cytokine elevation and the decline in cytokine levels after immunotherapy suggested an immune-inflammatory process accompanying the CIDP phase. Together with albuminocytologic dissociation, peripheral nerve enlargement, electrophysiological demyelination, negative CNS demyelinating antibody testing, and the absence of demyelinating lesions on brain MRI, these findings favored predominant peripheral nervous system involvement. Negative antibody testing does not exclude CIDP, particularly in children. The cytokine changes should therefore be interpreted as markers accompanying disease activity and treatment response, rather than evidence of a causal mechanism.

The marked elevation of CSF protein during the CIDP phase was probably multifactorial. In CIDP, elevated CSF protein may result from nerve root inflammation, blood–nerve or blood–CSF barrier dysfunction, inflammatory activation, complement involvement, and impaired protein clearance from affected nerve roots (18). In this patient, nerve root inflammation and barrier dysfunction may have contributed to protein leakage into the CSF; the elevated IgG index and 24-h intrathecal IgG synthesis suggested concurrent intrathecal immune activation. Thus, the CSF protein elevation was unlikely to be explained by intrathecal IgG synthesis alone (18). In this patient, CSF protein was only mildly increased during the septic phase but rose markedly during the CIDP phase, together with increased CSF albumin, elevated IgG index, and increased 24-h intrathecal IgG synthesis. Oligoclonal bands and serum immunoglobulins were normal. These findings suggest that the CSF protein elevation was related mainly to nerve root inflammation and barrier dysfunction, with concurrent intrathecal immune activation, rather than to intrathecal IgG synthesis alone. The subsequent decline in CSF protein after IVIg and corticosteroids further supported a treatment-responsive inflammatory component.

No disease-modifying treatment is currently available for the underlying PMP22-related hereditary demyelinating neuropathy with a severe Dejerine–Sottas-like phenotype. Recognition of a superimposed inflammatory neuropathy is clinically important. IVIg, corticosteroids, and plasma exchange remain established therapeutic options for CIDP, while biologic therapies targeting B cells, complement, or inflammatory pathways have been explored in selected refractory cases (4, 19, 20). In this patient, IVIg and high-dose corticosteroids were followed by improvements in consciousness, muscle strength, and respiratory function, together with reductions in serum cytokines and CSF IL-8. Recovery was incomplete, probably because of severe pre-existing hereditary neuropathy and secondary axonal injury. The slow decline in CSF protein and persistent disability after discharge suggest that inflammatory activity and chronic axonal damage both contributed to the outcome, supporting the need for long-term clinical and electrophysiological follow-up and individualized maintenance immunotherapy.

This report has several limitations. It describes a single patient, and the temporal association between influenza B infection, cytokine changes, and CIDP does not establish causality. Cytokine profiling was descriptive, and its mechanistic relevance remains uncertain. Some electrophysiological parameters, including conduction block and temporal dispersion, could not be assessed during the most severe phase because motor and sensory responses were absent. The genotype–phenotype correlation should also be interpreted with caution given the variable expressivity of the maternally inherited 17p12 deletion. In addition, although improvement after immunotherapy supports a treatable inflammatory component, the contributions of intensive care support, rehabilitation, and spontaneous recovery cannot be excluded. Nevertheless, acute or subacute neurological deterioration after infection in children with longstanding neuropathic features should prompt evaluation for superimposed CIDP, as the inflammatory component may be treatable and may reveal an underlying hereditary neuropathy.

## Data Availability

The datasets presented in this article are not readily available because of ethical and privacy restrictions. Requests to access the datasets should be directed to the corresponding author.
